# Dietary Habit Modifications in Paediatric Patients after One Year of Treatment with the Crohn’s Disease Exclusion Diet

**DOI:** 10.3390/nu15030554

**Published:** 2023-01-20

**Authors:** Rafael Martín-Masot, Marta Herrador-López, Víctor Manuel Navas-López

**Affiliations:** Paediatric Gastroenterology and Nutrition Unit, Hospital Regional Universitario de Málaga, 29010 Málaga, Spain

**Keywords:** Crohn’s disease, Crohn’s disease exclusion diet, dietary habits, children

## Abstract

Gut microbiota alterations play a key role in the pathogenesis of inflammatory bowel disease (IBD), and its modification through specific diets is an emerging line of treatment that is currently being researched. The aim of this study was to assess changes in the dietary habits of patients with Crohn’s disease (CD) and their long-term adherence to dietary therapy with the Crohn’s disease exclusion diet (CDED) after one year. To analyse the modification of dietary habits and adherence to the Mediterranean diet (DM), the KIDMED questionnaire, a food consumption frequency questionnaire, was used. Twenty-four hour recalls at two timepoints, namely prior to the start of the dietary therapy and one year later, were also carried out. The processing degrees of the foods consumed by the patients were analysed at both timepoints using the NOVA classification system. Data from 24 patients (15 boys), with a mean age of 12.7 ± 2.9 years at the start of the dietary therapy, were analysed. All patients showed an improvement in their dietary pattern in the form of a reduction in the intake of ultra-processed foods (UPFs); a higher adherence to the MD; a decrease in the intake of cold meats, seafood, pasta, precooked foods, soft drinks, and pastries; an increase in the intake of eggs, legumes, dairy products, and nuts (*p* < 0.05); and adequate adherence, even in the long-term, to foods allowed during the different phases of the dietary regimen. Although more factors have an impact on the course of the disease in these patients, improving their dietary habits is essential.

## 1. Introduction

Crohn’s disease (CD) is a chronic inflammatory disease characterized by involvement of any part of the gastrointestinal tract. It is a form of inflammatory bowel disease (IBD), a term which also comprises ulcerative colitis (UC) and unclassified inflammatory bowel disease (IBDU). The incidence of CD is increasing among paediatric patients worldwide parallelly to changes in the nutritional habits and westernisation of this age group’s diet [1]. These changes are considered key factors in the pathogenesis of the disease due to their plausible role in altering the gut microbiota, metabolome, host barrier function, and innate immunity [2].

Diet can modulate the gut microbiota due to its plasticity, and is currently considered to be an emerging line of treatment and research in CD. Dietary components that are red-flagged due to their ability to alter the microbiome are commonly found in the so-called “Western diet”, which is characterised by a high fat, sugar, wheat, and dairy content, as well as a decreased intake of natural dietary ingredients, in addition to a low exposure to fibre and a high exposure to multiple food additives [2]. The components and additives consumed in this diet are mainly contained in ultra-processed foods (UPFs), whose production and consumption has expanded worldwide in recent years. UPF are formulated using five or more industrial ingredients that are characterised by a high caloric content and low nutritional density; contain many additives; are rich in sodium, saturated fats, and simple sugars; are low in fibre, protein, and various micronutrients; and are, therefore, considered to be damaging to people’s health [3].

Several mechanisms have been related to epithelial barrier defects in patients with CD. On the one hand, the permeability is primarily increased, there is a greater exposure of bacterial antigens that will lead to a loss of tolerance and inflammation. On the other hand, the increase in intestinal permeability could lead to bacterial translocation, a situation that could develop an adaptive immune response that aims to contain the process. Furthermore, certain components of the diet, generally those contained in a westernized diet capable of altering that mechanism involved in the control of intestinal permeability, could stimulate bacterial adherence to the intestinal epithelium and leads to stimulation of the immune system and produce inflammation [2,4]. Therefore, diet is an important challenge to improve these mechanisms. In fact, diet does not only impact on the microbiome, but also impacts on host barrier and immunity. Dietary changes and changes in the gastrointestinal microbiota can modify the production of different metabolites that participate in the fermentation of various components, producing, among others, short-chain fatty acids, key in maintaining intestinal permeability [5]. The alteration of intestinal permeability allows the passage of antigens to the systemic circulation and the activation of the immune system [6]. In addition, gastrointestinal microbiota produces trophic stimulation on the enterocyte, helping to maintain the integrity of the intestinal barrier or stimulating peristalsis. At the host defence level, the microbiota participates in inhibiting the adhesion of different pathogens [7,8], secretes antimicrobial components such as lactate, induces the synthesis of antimicrobial proteins such as defensins, and stimulates IgA secretion.

Exclusive enteral nutrition (EEN) is being used for over 30 years as a dietary therapy to induce clinical and endoscopic remission in paediatric CD [9]. However, in recent times, the Crohn’s disease exclusion diet (CDED) is being used for the same purpose. This diet is based on the elimination of certain dietary components that might have an impact on gut microbiota, inflammatory response, and intestinal permeability. This dietary therapy has not only proven to be as effective as EEN in inducing remission in both newly diagnosed paediatric CD patients and those who have exhibited a loss of response to anti-tumour necrosis factor (TNF) therapy [10,11,12], but it has also been seen to be superior to EEN in terms of tolerance and compliance [12]. It allows for overcoming the difficulties posed by EEN and represents an advancement with respect to partial enteral nutrition in the treatment of CD, as it allows access to food, albeit in a standardised and controlled manner. Although it is known that it can be used as an induction strategy, it is important demonstrate if CDEC could also be an option for long-term sustained remission, more tolerable and with more compliance for patients.

Because of the perceived importance of the role played by nutrition in this disease, a key strategy in the treatment of CD is to improve the dietary habits of children who suffer from this condition. Thus, information must be collected from patients who have followed a CDED for a long time in order to draw more solid conclusions about its importance and determine whether the guidelines provided within a nutrition education programme are sustained over the long-term, as this could have an impact on the prognosis of the disease in adulthood. The main objective of this study was to assess long-term adherence to the CDED and the changes in the dietary habits of CD patients consuming this diet after one year of follow-up.

## 2. Materials and Methods

### 2.1. Subjects

The study was carried out according to the principles of the Declaration of Helsinki and approved by the Ethics Committee of the Hospital Regional Universitario of Málaga (N:1294-N-16). Patients aged 8–18 years old with established diagnosis of CD (according to Porto Group criteria [13] who signed the informed consent, were prospectively included. Patients who had active extra intestinal disease, patients with complicated disease (structuring or penetrating disease), or those who had active perianal disease (active fistula or abscess) were excluded.

### 2.2. Clinical and Socio-Demographics

Participants’ clinical and socio-demographic characteristics were assessed by the same group of researchers. Anthropometric characteristics (weight, height) of the participants were measured under the same conditions using a stadiometer (Seca 22, Hamburg, Germany).

To evaluate mucosal inflammation, we use the MINI index (Mucosal Inflammation Non Invasive), that allows a non-invasive evaluation of mucosal inflammation in children with CD, identifying with high sensitivity and specificity [14] those presenting mucosal healing (MINI index < 8).

### 2.3. Dietary Assessment

Dietary intake and habits data were collected from paediatric patients at study baseline and after 52 weeks following a CDED.

A minimum of four on-site visits (at diagnosis and at weeks 6, 13, 24, and 52) and three telephone check-ups calls (at weeks 3, 9, and 18) to assess dietary compliance were performed with the team’s dietitian throughout the year of follow-up. Telephone contact was attempted up to three different times if they did not answer to the first call. These contacts involved a complete nutritional assessment, a review of the patients’ dietary history, an explanation of the different phases of the CDED, a reinforcement of nutritional education concepts according to the needs of each patient, and a review of their adherence to the dietary regimen. Participants were provided with a telephone number and an email address in case they had any dietary questions between visits.

At the two study timepoints, the patients’ macro- and micro-nutrient intakes were also analysed based on an assessment of two 24-h dietary recalls. This assessment was conducted using Odimet, an online nutritional calculation tool which allows the estimation of macronutrients and micronutrients.

Regarding the dietary habits, we asked them to fill out a short questionnaire at week 0 and week 52 which included questions on the portions consumed of the main food groups (fruits, vegetables, meat, sausages, fish, seafood, eggs, legumes, dairy products, rice, pasta, potatoes, nuts, precooked foods, soft drinks, sweets and pastries) with the following options available: Never, 1–3 servings/month, 1 serving/week, 2–4 servings/week, 5–6 servings/week, 1 serving/day, 2–3 servings/day, 4–6 servings/day, or 6+ servings/day.

Changes in the subjects’ dietary habits were analysed based on the KIDMED Mediterranean diet (MD) adherence questionnaire score, the processing grade of the foodstuffs contained in the 24-h recalls, and a 140-item food frequency questionnaire. The information sourced from this last instrument was also used to assess adherence to the dietary regimen. The KIDMED questionnaire [15] includes 16 questions that can be scored, and scores are added up to quantify the total index of the subject’s adherence to the MD. The KIDMED index ranges from 4 (no adherence to the MD) to 12 (complete adherence to the MD) (≥8 equivalent to an optimal Mediterranean diet, 4–7 equivalent to a need for dietary pattern improvements, and ≤3 equivalent to a very poor-quality diet).

On the other hand, the processing degrees of the foods consumed by the participants was analysed using the NOVA classification system, which divides foods into four groups based on their processing level (group 1: unprocessed or minimally processed foods; group 2: processed culinary ingredients; group 3: processed foods; and group 4: UPFs). 

### 2.4. Data Analyses

A descriptive study of the sample and the variables was conducted, presenting the data as mean values plus their standard deviation. The Chi-square test or Fisher’s test were used to analyse qualitative variables. Student’s *t*-test for independent samples was used to compare means when the sample’s normality and equality of variances had been verified; otherwise, the Mann–Whitney test or Welch’s test were used. A *p*-value < 0.05 was considered statistically significant.

## 3. Results

Data were obtained from 24 patients (15 boys) with a mean age of 12.7 ± 2.9 years at the start of the dietary treatment. There were no dropouts among the participants. The clinical characteristics of these patients and the classification of their disease [14] are listed in Table 1.

An analysis of the 24-h dietary recall at study baseline and after 52 weeks of treatment showed statistically significant differences in the percentage of energy obtained from UPFs, as well as in the intake of protein, fibre, and iron (Table 2).

There were no significant differences in the percentage of energy obtained from the main macronutrients at the two analysed timepoints, with protein intake exceeding the recommended intake for the subjects’ age on both occasions.

The KIDMED index of adherence to the MD also showed significant differences, with the mean score increasing from 5 ± 2.1 to 7.5 ± 1.4 (Table 2). After 52 weeks of treatment, no patient had a very poor-quality diet compared with 33.33% of those who did at baseline. Moreover, the percentage of patients who followed an optimal MD increased by 37.5% after one year consuming the CDED.

**Figure 1:**
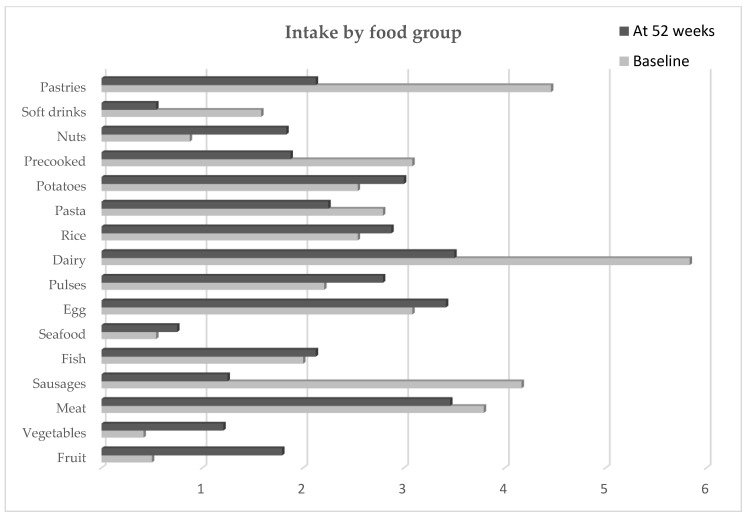
Intake by food group. Data are shown as medium value of servings/week, excepting fruit and vegetables, servings/day.

In terms of food consumption patterns, a statistically significant decrease was observed in the consumption of cold meats, seafood, pasta, precooked foods, soft drinks, and pastries, together with an increase in the consumption of eggs, legumes, dairy products, and nuts (*p* < 0.05) (Table 3, Figure 1). There were no changes in the consumption of meat, fish, potatoes, or rice. All analysed patients increased their mean daily intake of fruits and vegetables (fruits: 0.5 servings vs. 1.79 servings and vegetables: 0.42 vs. 1.21 servings). When asked about the type of fat used at home, all of our patients stated the use of olive oil as the main cooking fat in both 0 and 52 weeks, being this fact an assured point (+1) in the KIDMED questionnaire.

## 4. Discussion

This is the first study available analysing the dietary pattern of patients with paediatric CD treated with CDED in our setting and highlighting a major change with respect to the dietary pattern of these subjects prior to the diagnosis of their disease in terms of the consumption of fruit, vegetables, fibre, and iron.

Our patients exhibited an improvement in the dietary quality indexes, including a lower consumption of UPFs and a considerable reduction in the percentage of energy sourced from these products. A year after starting the therapy, these products mainly consisted in pastries and precooked foods that were included in the free foods allowed in this dietary regimen. Unhealthy dietary habits and the consumption of UPFs have been linked to the increase in chronic, non-communicable diseases and, specifically, digestive disorders, due to the changes that they induce in the gut microbiota, the body’s inflammatory response, and intestinal permeability. Several studies have correlated the consumption of UPFs with the increased incidence of chronic, non-communicable diseases in children and adults [16,17,18], identifying certain components and specific additives included by the industry in these foods as the cause of alterations in the barrier function, the immunity, the microbiome, and the increased intestinal permeability in CD [2]. This is why, in recent years, the epidemiological changes that affect CD have highlighted the importance of diet on the onset of this disease [19]. Therefore, it can be hypothesised that improvements in these patients’ dietary patterns can play an important role, both at the beginning and during the follow-up of their disease, with further clarifications being needed with regard to their role in the onset of this condition [2].

On the other hand, we observed a significative improvement in the clinical parameters at 52 weeks (*p* < 0.05 for C-reactive protein, erythrocyte sedimentation rate, daily stools, and MINI index). In our cohort, the patients had concomitant treatment and several factors contributed to the improvement. Although MINI index relies greatly on faecal calprotectin and perhaps overestimates the actual situation of the intestinal mucosa, it is a non-invasive tool that allows to evaluate the situation of the intestinal mucosa. However, it cannot be used exclusively for decision making in the follow-up of patients.

It is important to highlight that CDED is a therapeutic measure that is almost exempt of side effects. In our study, no patient had to discontinue the diet after one year of follow-up. In the maintenance phase of the diet, the aim for the patient is to acquire dietary habits favourable to their disease, given the previously emphasised importance of diet and its influence on the gut and the microbiome. Other measures, such as EEN, can only be used for short periods, usually in the induction phase due to their problems of adherence [9]. In fact, even in the induction phase, sometimes it requires the administration of the formula by artificial nutrition device (nasogastric tube) or even discontinuation, a situation that did not occur in our study with CDED.

The MD has proven to have positive effects on chronic diseases and has been recognised for its potential benefits in inflammatory bowel disease (IBD) [20]. In our case series, we observed a low adherence to the MD at diagnosis, which subsequently improved after administering the nutritional education programme. Maintenance of the CDED resulted in improvements in the quality of the patients’ diet, as demonstrated by the KIDMED index, with the figures obtained before starting this dietary therapy being similar to those reported in a previous study involving children with IBD and healthy controls [21]. Recently, Lewis et al. have reported the use of MD in adults with CD [22], suggesting that MD may be beneficial in mild and moderate disease, being superior to other dietary approaches.

Regarding the type of fat included on the diet, all our patients used olive oil in both studied moments, being this the recommended fat within the CDED and one of the main fat sources in the MD and in our country [15].

Because milk is among the restricted foods in the CDED (except for free meals), we also observed a considerable reduction in dairy intake. Despite this, it should be noted that 79.1% of the patients maintained a mean intake of 500 mL of a Modulen IBD^®^, a specific polymeric formula, owing to which no significant differences were observed in their mean calcium intake.

Although the number of weekly meat servings remained unchanged at the two study timepoints analysed, it is important to describe the source of these meat servings. After one year following the CDED, 100% of the patients maintained a high intake of chicken breast (2–4 servings per week) and 79.1% consumed one serving of beef per week (allowed as of the second phase of the CDED). Almost one third of the participants (29.1%) reported eating other lean meats, such as turkey or rabbit (1–3 portions per month), and 54.1% reported eating the same number of pork servings per month, while the remaining percentage of patients did not include pork in any meal throughout the month. Since red meat is one of the main foods that should be excluded in the CDED [4], and considering that 62.5% of the participants ate red meat at least once a week at study baseline, we can consider this drop in red meat intake one year later as a sign of adaptation to the dietary treatment.

A reduced consumption of dietary fibre has also been identified in different epidemiological studies as a potential risk factor for the onset of this disease [23,24], given that it can affect host immunity through multiple pathways. In our case series, fibre intake was still low compared with the general recommendations, although it was found to be similar to that reported in a previous study performed on children with CD [25] and in line with the mean intake pattern of Spanish children [26]. Comparable results were observed in a recent systematic review in adults with IBD whose fibre intake was higher than among our patients, but still below the recommended intake (25–30 g/day) [27].

## 5. Strengths and Limitations

There are several limitations that should be highlighted. Firstly, although further follow-up of the cohort is necessary to establish the sustainability of the diet, its beneficial effects (due to its probable anti-inflammatory potential and the composition of its components), are clear [10,12,28]; however, it is necessary to adapt the diet to the patient’s dietary habits. Nevertheless, we present the first follow-up at 52 weeks of this nutritional intervention, which suggests that the patients have integrated CDED into their dietary habits.

Secondly, another limitation of this study is that the dietary assessment was performed through a 24-h recall. Nonetheless, this is the first study available analysing the dietary habits of a non-negligible number of paediatric CD patients in our setting.

## 6. Conclusions

After one year following a CDED, all patients improved their adherence to the MD and drastically reduced their consumption of UPFs, with unprocessed or minimally processed foods making up more than half of their daily food intake. The participants demonstrated adequate adherence to the dietary regimen, obtaining their protein from the required foods included in the CDED (mainly chicken breast, eggs, and a polymeric formula) and reporting a significant reduction in the consumption of the main products excluded from this dietary regimen (red meat, cold meats, dairy, soft drinks, and UPFs). Their dietary pattern also improved, with an increase in the intake of key foods that modulate the gut microbiota included throughout the different phases of the CDED. Although there are more factors influencing the course of this disease in these patients, improving their dietary habits is essential. To this end, maintaining a CDED under the supervision of a specialised dietitian is deemed an effective strategy. Further studies with a larger number of patients must be performed to confirm our conclusions.

## Figures and Tables

**Table 1:** Clinical characteristics of the patients included in the study.

Variable	Crohn’s Disease (*n* = 24)	
**Age (years)**	12.7 (2.9)	
**Age at diagnosis**	11.1 (2.4)	
**Sex, male (%)**	15 (62.5)	
	**Baseline**	**At 52 weeks**
**Weight (kg)**	41.8 (13.3)	46.9 (13.3)
**Height (cm)**	149.3 (14.5)	153.9(14.6)
**BMI (kg/m^2^)**	18.2 (3.7)	19.4 (3.5)
**Clinical data**	**Baseline**	**At 52 weeks**
*C-reactive protein (mg/L) (median value)*	9.10 (IQR: 2.9–51.3)	4 (IQR: 2.9–4)
*Erythrocyte sedimentation rate (mm/h)*	25.93 (17.3)	13.8 (10.4)
*Daily stools*	2.7 (2.1)	1.5 (0.6)
*Faecal calprotectin (μg/g) (median value)*	1111 (IQR: 448.1–2375.5)	512 (IQR: 58.1–976.4)
*MINI index*	14.1 (5.1)	9 (5.98)
**IBD classification**		
**Age at diagnosis**		
*A1a (%)*	4 (16.7)	
*A1b (%)*	20 (83.3)	
**Location**		
*L1 (%)*	10 (41.7)	
*L2 (%)*	1 (4.2)	
*L3 (%)*	7 (29.2)	
*L4a (%)*	1 (4.2)	
*L3 + L4 (%)*	5 (20.7)	
**Behaviour**		
*B1 (%)*	23 (95.8)	
*B3 (%)*	1 (4.2)	
*p (%)*	2 (8.4)	
**Growth impairment**		
*G0 (%)*	20 (83.3)	
*G1 (%)*	4 (16.7)	
**Extraintestinal manifestations**		
**Erythema nodosum (%)**	2 (8.33)	

A1a: 0–9 years; A1b: 10–16 years; L1: distal third of the ileum and caecum; L2: colon; L3: ileocolon, L4a: upper disease proximal to the ligament of Treitz; B1: non-stenosing and non-penetrating; B3: penetrating; *p*: perianal disease; G0: no evidence of growth retardation; G1: growth retardation. Unless otherwise stated, values are shown as a mean (standard deviation). IQR: Interquartile range.

**Table 2:** Differences in dietary habits upon starting the CDED and at 52 weeks.

	Baseline (*n* = 24)	At 52 Weeks (*n* = 24)	*p*
**Energy (kcal/day)**	1613.49 (360.5)	1794 (383.3)	0.09
**TEE covered (%)**	84.43 (18)	90.59 (21.6)	0.29
**NOVA classification**			
*Unprocessed or minimally processed (%E)*	34.49 (14.1)	56.41 (11.2)	<0.01
*Processed culinary ingredients (%E)*	15.72 (6.6)	22.16 (8.8)	<0.01
*Processed foods (%E)*	13.56 (11)	17.93 (10.6)	0.16
*Ultra-processed foods (%E)*	36 (19.1)	2.61 (5.2)	<0.01
**Adherence to the Mediterranean diet (KIDMED)**	5 (2.1)	7.5 (1.4)	<0.01
**Proteins (%)**	17.8 (3.8)	19.9 (2.8)	0.03
**Proteins (g)**	70.43 (17.4)	88.19 (18.6)	<0.01
**Carbohydrates (%)**	38.3 (6.4)	36.6 (6.1)	0.36
**Carbohydrates (g)**	180.1 (54.6)	195.3 (43)	0.5
**Fats (%)**	44 (7.8)	43.4 (5.9)	0.78
**Fats (g)**	68.7 (20.4)	73 (23.9)	0.29
**Fibre (g)**	6.1 (4.5)	10.5 (6.8)	0.01
**Calcium (mg)**	733.1 (293.8)	686.8 (273.1)	0.57
**Iron (mg)**	6.71 (2.5)	11.2 (3.5)	<0.01

E: energy; TEE: total energy expenditure. Data are presented as a mean (standard deviation).

**Table 3:** Intake by food group upon starting the CDED and at 52 weeks.

Food	Number of Servings	Patients, No. (%)		*p*-Value
		Baseline	At 52 Weeks	
**Fruit (servings/d)**	0	13 (54.2)	3 (12.5)	<0.0001
	1	10 (41.7)	6 (25)	
	2	1 (4.2)	9 (37.5)	
	3	0 (0)	5 (20.8)	
	4	0 (0)	1 (4.2)	
**Vegetables (servings/d)**	0	14 (58.3)	2 (8.3)	<0.0001
	1	10 (41.7)	15 (62.5)	
	2	0 (0)	7 (29.2)	
**Meat (servings/wk)**	2–4	10 (41.7)	14 (58.3)	0.101
	5–6	9 (37.5)	9 (37.5)	
	≥7	5 (20.8)	1 (4.2)	
**Sausages (servings/wk)**	0	2 (8.3)	14 (58.3)	<0.0001
	1	1 (4.2)	4 (16.7)	
	2–4	5 (20.8)	4 (16.7)	
	5–6	3 (12.5)	2 (8.3)	
	1/d	7 (29.2)	0 (0)	
	2–3/d	6 (25)	0 (0)	
**Fish (servings/wk)**	0	3 (12.5)	1 (4.2)	0.341
	<1	3 (12.5)	1 (4.2)	
	1	9 (37.5)	16 (66.7)	
	2–4	9 (37.5)	6 (25)	
**Seafood (servings/wk)**	0	15 (62.5)	8 (33.3)	0.037
	<1	7 (29.2)	14 (58.3)	
	1	0 (0)	2 (8.3)	
	2–4	2 (8.3)	0 (0)	
**Egg (servings/wk)**	1	3 (12.5)	1 (4.2)	0.036
	2–4	18 (75)	13 (54.2)	
	5–6	1 (4.2)	9 (37.5)	
	≥7	2 (8.3)	1 (4.2)	
**Pulses (servings/wk)**	0	2 (8.3)	0 (0)	0.011
	<1	2 (8.3)	1 (4.2)	
	1	9 (37.5)	3 (12.5)	
	2–4	11 (45.8)	20 (83.3)	
**Dairy (servings/d/wk)**	1/wk	1 (4.2)	2 (8.3)	<0.0001
	2–4/wk	0 (0)	15 (62.5)	
	5–6/wk	0 (0)	1 (4.2)	
	1/d	4 (16.7)	5 (20.8)	
	2–3/d	15 (62.5)	1 (4.2)	
	4–6/day	4 (16.7)	0 (0)	
**Rice (servings/wk)**	0	2 (8.3)	0 (0)	0.106
	1	5 (20.8)	4 (16.7)	
	2–4	17 (70.8)	19 (79.2)	
	5–6	0 (0)	1 (4.2)	
**Pasta (servings/wk)**	<1	0 (0)	4 (16.7)	0.004
	1	5 (20.8)	10 (41.7)	
	2–4	19 (79.2)	10 (41.7)	
**Potatoes (servings/wk)**	0	1 (4.2)	0 (0)	0.022
	<1	1 (4.2)	0 (0)	
	1	7 (29.2)	2 (8.3)	
	2–4	14 (58.3)	20 (83.3)	
	5–6	1 (4.2)	2 (8.3)	
**Precooked (servings/wk)**	0	0 (0)	1 (4.2)	<0.0001
	<1	2 (8.3)	3 (12.5)	
	1	2 (8.3)	18 (75)	
	2–4	13 (54.2)	2 (8.3)	
	5–6	6 (25)	0 (0)	
	≥7	1 (4.2)	0 (0)	
**Nuts (servings/wk)**	0	13 (54.2)	4 (16.7)	0.009
	<1	6 (25)	7 (29.2)	
	1	5 (20.8)	2 (8.3)	
	2–4	0 (0)	11 (45.8)	
**Soft drinks (servings/wk)**	0	5 (20.8)	15 (62.5)	0.001
	<1	6 (25)	5 (20.8)	
	1	7 (29.2)	4 (16.7)	
	2–4	6 (25)	0 (0)	
**Pastries (servings/wk)**	<1	0 (0)	4 (16.7)	<0.0001
	1	1 (4.2)	15 (62.5)	
	2–4	7 (29.2)	4 (16.7)	
	5–6	2 (8.3)	0 (0)	
	1/d	8 (33.3)	1 (4.2)	
	2–3/d	6 (25)	0 (0)	

wk: week, d: day.

## Data Availability

The data presented in this study are available in the article.
